# Supplementary material for: The Chagas non-endemic (ChaNoE) cohort: Aims and study protocol
Source: PLoS One. 2025 Apr 15;20(4):e0320637. doi: 10.1371/journal.pone.0320637 (PMC11999160; doi:10.1371/journal.pone.0320637)
Supplement: Attachment — Submitted filename: Plos_one_review.pdf [file pone.0320637.s001.pdf]

Dear Editor and Authors,

Thank you for the opportunity to review the manuscript titled "The Chagas Non-Endemic Cohort (“ChaNoE”): Aims and Study Protocol." This is an intriguing and valuable project. The manuscript effectively describes the establishment of a Spanish cohort designed to identify and monitor chronic cases of Chagas disease. The study employs a modern clinical-laboratory approach and clearly articulates its objectives and rationale in a well-justified manner. The manuscript addresses an important public health challenge: Chagas disease in non-endemic regions, a topic that remains underrepresented in current research. The focus on Spain—a country with a significant population of individuals living with Chagas disease due to migration—highlights the study’s relevance and potential impact. However, before the manuscript is accepted, several questions and concerns need to be addressed, as outlined below:

Major comments

1. The authors mention multiple times that the data obtained from the “ChaNoE” cohort will be compared with other cohorts worldwide. However, the manuscript does not specify these cohorts. Including examples of similar studies, such as Oxente Chagas Bahia, Samitrop, TESEU, and others, would enhance the manuscript by informing readers of relevant ongoing research efforts globally and providing context for the comparative analyses planned in this study.
2. While the protocol is thorough, some methodological aspects, such as the specific strategies for data standardization across centers, are underexplained. A clearer description of quality control for data and biobank management is needed.
3. The study’s reliance on multiple centers for participant recruitment could face logistical hurdles, especially in ensuring consistent diagnostic criteria and follow-up. Comment!
4. Although PCR is mentioned, its limited sensitivity in chronic cases raises concerns. The manuscript could better address how it will account for false negatives or inconsistent results. Explain how the study will mitigate the limitations of current diagnostic tools, such as PCR, and whether alternative or complementary methods will be employed.
5. While the protocol outlines periodic evaluations, the manuscript does not provide sufficient detail on how adherence to follow-up will be ensured, particularly for participants in non-urban areas. Discuss strategies to overcome potential challenges in participant recruitment and retention, including communication methods and incentives.
6. Inclusion criteria requiring diagnosis by two serological tests might exclude some cases, potentially limiting generalizability.
7. Add visual elements, such as flowcharts or timelines, to illustrate the study design, recruitment process, and follow-up schedule.
8. The potential financial burden of implementing such protocols in other settings, including low-resource regions, is not addressed. Include a discussion of how the findings and methodologies could be adapted for low-resource or other non-endemic settings. In addition, provide examples of how the study outcomes will directly influence public health policies or clinical practices in non-endemic areas.
9. Include plans for biobank management and data sharing policies.

Minor comments

1. Line 44: Italicize *Trypanosoma cruzi*.
2. Line 49: Clarify the term "heterogeneous." As it stands, the meaning is unclear and could benefit from further explanation or rephrasing?
3. Line 81: Remove the word "also." The phrase, as written, might imply that vectorial transmission occurs in non-endemic areas, which could lead to misinterpretation.
4. Line 403: Specify the microbiological tests used for diagnosing Chagas disease. Wouldn't these be more accurately described as parasitological tests?
5. Line 470: Capitalize "Chagas."
