# Supplementary material for: The Chagas non-endemic (ChaNoE) cohort: Aims and study protocol
Source: PLoS One. 2025 Apr 15;20(4):e0320637. doi: 10.1371/journal.pone.0320637 (PMC11999160; doi:10.1371/journal.pone.0320637)
Supplement: Attachment — Submitted filename: Response to Reviewers.docx [file pone.0320637.s003.docx]

**Response to Reviewers:**

**Reviewer #1: The authors propose a multi-center study cohort to investigate Chagas disease in a non-endemic country. This is a very important initiative and I congratulate the authors.**

**Comments:
1) Please, include in the objectives the study of clinical end-points. Hard end-points, such as all-cause mortality and heart transplant, but also secondary end-points, such as, admission due to heart failure, cardiac device implantation, and stroke. As a lot of data will be collected, it is very important to understand how these variables are associated with clinical outcomes.**

Thank you for your valuable comment. Upon reviewing our manuscript, we realized that we had omitted a detailed description of the clinical and laboratory outcomes, as well as the criteria for their measurement and definition. Therefore, we have now included both primary and secondary outcomes in the "Aims and Objectives" section. In accordance with the reviewer’s suggestion, we have specified the criteria for disease progression in both the cardiac and digestive forms and clarified the definition of cure.

**2) Please, clarify criteria for disease progression. Do you mean only progression from indeterminate to cardiac form (A to B/C or D) ? Or also progression within stages of the cardiac form (B to C or D)? Will also study progression from indeterminate to digestive form?**

Please, refer to our response to your previous comment.

**3) Please, clarify the criteria for cure that will be followed.**

Please, refer to our response to your previous comment.

**4) Please, clarify if a patient will be classified as B if changes are present in the echocardiogram even in the presence of a normal ECG. I ask this because of the following sentence: “CCC will be defined by the presence of segmental or global left ventricle (LV) or right ventricle (RV) systolic function impairment, increased end-diastolic diameter of the LV (>55mm), ventricular aneurysms or cardiac thrombus”. Usually, an abnormal ECG is always necessary for a patient to be diagnosed with CCC.**

Although this scenario is infrequent, different authors have proposed that echocardiography can also detect early alterations in patients with normal ECG findings*. While this approach may not be feasible in all settings, we believe that such alterations should be considered, as these patients are likely at higher risk of further progression compared to those with completely normal findings. Further evidence is needed in this regard, and we hope that the present cohort will contribute valuable data. We acknowledge that this may pose challenges in comparing our results with other cohorts; however, we plan to conduct subgroup analyses for these patients.

To ensure clarity, we have modified the relevant sentence to: “CCC will be defined by the presence of segmental or global left ventricle (LV) or right ventricle (RV) systolic function impairment, increased end-diastolic diameter of the LV (>55mm), ventricular aneurysms, or cardiac thrombus, even in the presence of a normal ECG.” Additionally, we have introduced the quotation “^2^” to “Table 2 Classification and follow-up of…”

* Laynez-Roldán P, Losada-Galván I, Posada E, Ávila L de la T, Casellas A, Sanz S, et al. Characterization of Latin American migrants at risk for Trypanosoma cruzi infection in a non-endemic setting. Insights into initial evaluation of cardiac and digestive involvement. PLoS Negl Trop Dis 2023;17. https://doi.org/10.1371/JOURNAL.PNTD.0011330.

* Ralston K, Zaidel E, Acquatella H, Barbosa MM, Narula J, Nakagama Y, et al. WHF Recommendations for the Use of Echocardiography in Chagas Disease. Glob Heart 2023;18. <https://doi.org/10.5334/GH.1207>.

**5) Table 2. A 24-Holter is always necessary in the initial evaluation of all patients with CCC. Specially if the study objective is the evaluation of prognosis1.**

We anticipated this comment, as it has been a point of discussion among infectious disease specialists, cardiologists, and professionals working in less complex institutions. While we acknowledge the limitation of not performing a baseline Holter in all patients, we ultimately decided to conduct Holter monitoring in all patients classified as CCC (from stage A to D), as well as in those with normal ECG but abnormal echocardiographic findings or suggestive symptoms, as specified in Table 2. This decision was made after considering the current lack of strong evidence supporting the cost-effectiveness of a 24-hour Holter in asymptomatic patients with normal ECG and echocardiographic findings.

**6) Please, evaluate the inclusion of biochemistry and blood count in the initial evaluation and follow-up. A poor kidney function, anemia, low sodium are all associated with a dismal outcome in patients with heart failure.**

We fully agree with the reviewer that these parameters are essential at inclusion. Consequently, we have incorporated biochemical and hematological assessments into the initial evaluation. In follow-up, we decided to maintain it optional depending on each case (many of our patients are young individuals with no comorbidities and sometimes they already have other blood tests performed by their family doctor.

**7) Table 2. Echocardiography in stage A (indeterminate) can be at every 5 years. B1 patients should undergo echocardiograms more frequently as they already have changes in ECG: every two years, and B2 and C patients every year1.**

Thank you for your suggestion. We have adjusted the follow-up intervals for B1 patients accordingly. The follow-up periods for B2 and C patients were already in line with the recommendation.

**8) Table 6. Correct fifth line “5mg/day (bid or tid)”**

The correction has been made.

**9) Benznidazole treatment dose recommendation is different in Brazil. “For adults with chronic CD, benznidazole is administered orally at the dosage of 5mg/kg/day divided into two or three doses, for 60 days, with a recommended maximum dosage of 300mg/day. For individuals with acute CD, the dose can be of as much as 10mg/kg/day. For individuals weighing over 60 kg, the therapeutic schedule can be extended to achieve the ideal target dose, maintaining 300 mg as the daily limit to prevent adverse events. The 300mg benznidazole regimen can be used for the number of days equivalent to the individual’s weight, limited to a total of 80 days even for individuals weighing over 80 kg”2. The author’s table 6 suggests that there is no upper limit for benznidazole daily dose. For example, a patient with 90 kg would be prescribed 450 mg/day but this can increase the risk of adverse effects.**

In Spain, we have traditionally used weight-adjusted dosing, extending treatment only in specific cases. Following discussions with participating centers, we found that while a few centers have used up to 400mg/day, most adhere to the 300mg/day limit when weight does not exceed 100kg. To clarify this, we have added a note after Table 6.

Regarding adverse events, the literature presents conflicting data on the relationship between dosage and toxicity. Notably, a recently published clinical trial (The Multibenz Trial, PMID: 38218195) employed a 400mg/day dosage without observing a significant increase in adverse events.

**10) Include in all Tables the references the authors used.**

We have now included references in all tables except Table 7, which is based on our own consensus derived from the available evidence.

**11) Statistical analysis. Include how prognosis will be evaluated. Survival curves, logistic regression, non-adjusted or adjusted, ROC curves? Please, clarify**

To address this, we have added a paragraph to the "Data Management and Statistical Analysis" section detailing how we will analyze survival outcomes and potential confounders.

**12) Dissemination plan. Is there a plan to make data available in public repositories?**

As approved by the Ethics Committee, data will be shared with appropriate academic parties upon request to the chief investigator. We have added a paragraph clarifying this in the "Ethical Considerations and Dissemination Plans" section.

**13) Discussion. There are other papers describing guides for the generation of databases or repositories for Chagas disease cohorts. I invite the authors to read those papers3,4.
1 - Saraiva RM, Mediano MFF, Mendes FS, Sperandio da Silva GM, Veloso HH, et al. Chagas heart disease: An overview of diagnosis, manifestations, treatment, and care. World J Cardiol. 2021 Dec 26;13(12):654-675.**

**2 - Marin-Neto JA, Rassi A Jr, Oliveira GMM, Correia LCL, Ramos Júnior AN, et al. SBC Guideline on the Diagnosis and Treatment of Patients with Cardiomyopathy of Chagas Disease - 2023. Arq Bras Cardiol. 2023 Jun 26;120(6):e20230269.**

**3-González Martínez A, Losada-Galván I, Gabaldón-Figueira JC, Martínez-Peinado N, Saraiva RM, et al. A standardized clinical database for research in Chagas disease: The NHEPACHA network. PLoS Negl Trop Dis. 2024 Aug 15;18(8):e0012364.**

**4-Martínez-Peinado N, Gabaldón-Figueira JC, Rodrigues Ferreira R, Carmen Thomas M, López MC, et al. A guide for the generation of repositories of clinical samples for research on Chagas disease. PLoS Negl Trop Dis. 2024 Aug 15;18(8):e0012166.**

Thank you for this suggestion. Some of our investigators have already participated in developing consensus guidelines to harmonize and facilitate comparability in prospective Chagas disease studies. Recognizing the importance of this issue, we have incorporated the suggested references and added relevant sentences to the third paragraph of the Discussion.

**Reviewer #2: Summary:**

**This article describes the literature behind, and some of what the Spanish Chagas non-endemic cohort (ChaNoE), a multi-center initiative based in Spain, seeks to accomplish. Their aim is to improve the understanding and management of Chagas disease (CD) in non-endemic regions, and to contribute evidence regarding clinical progression, biomarkers, and overall understanding of Chagas disease. The study focuses on: They aim to recruit individuals with chronic CD, standardize diagnostic and treatment protocols, monitor disease progression, and create a biobank to facilitate biomarker research for progression and treatment efficacy. A serum biobank is being established to identify and validate biomarkers for disease progression and treatment response. This is a critical need in CD research. The Spanish experience is useful for cohorts elsewhere in non-endemic areas. The authors do a good job reviewing existing literature on CD, including its classification and management. However, the article reads more like a review of literature rather than a research study protocol. There are many details missing from this description, such as inclusion and exclusion criteria, detailed timelines, where the samples will be taken and stored, which specific serology tests will be used to diagnose CD, who will recruit participants, etc. This is a great idea for a project, and the achievement of the cohort will be very beneficial. However, in order to publish the protocol, it needs to provide much more detail.**

Thank you for all your comments. We really appreciate them and think that the following changes will improve the article’s quality. We will answer the specific questions below.

**Introduction:
• In line 92, it says that treatment is recommended for people in the chronic phase. This is not exactly accurate. The recommendations depend on the age of the individual and their circumstances, as the BENEFIT trial showed that treatment in older individuals with advanced cardiomyopathy did not result in significant improvement. Treatment is more strongly recommended for younger patients. However, guidelines vary by region. Please be specific about the treatment indications for chronic disease in Spain.**

Thank you for your comments. As you mentioned, there is an ongoing debate on treatment benefit and indications. There are several controversies and evidence in favor and against treating chronic patients, especially those with advanced age and mild cardiopathy. The BENEFIT trial showed us that in moderate or advanced cardiomyopathy, parasitological treatment offers no effect on disease progression, but in other groups we consider the evidence provided by this study as limited. However, this evidence has been widely debated, particularly regarding its applicability to all chronic patients. During last years, many countries have changed their protocols in favor of being laxer in its indication, and for example, Brazil has started to treat much more patients during last years.

Referring to age limits, we consider that the habitual 50-year-age limit has been based on limited life expectancy in most endemic regions. However, these populations, when migrating, have showed a tendency to equal the native population which in Spain the mean is around 83 years old. In Spain we already have much experience treating elder patients and we are close to publish our results supporting this indication during the present year. In summary, considering the available evidence and our own experience, we have set our indications in Table 7.

**• The introduction should typically end with the overall aim of the study/work. In this case, it could be more specific about the particular context in Spain and state that the article will describe the cohort and its intent.**

We appreciate your suggestion. We have revised the final paragraph of the introduction to explicitly state the study’s objectives within the Spanish context, ensuring alignment with standard scientific structuring.

**Methods:
• Overall, a large proportion of the paragraphs in the methods section seem more like introductory paragraphs, describing the literature. Please remember that the introduction or background section of an article reviews existing literature, and this often happens again in the discussion section when comparing findings to existing studies. Please reorganize the article to clarify each section. The methods should describe the project itself rather than the literature.**

Thank you for this comment. We completely agree with you that the previous structure seems more like a literature review with an embedded protocol. In this sense, we have moved some of the information to the discussion, maintaining only those paragraphs that are essential to understand the protocol and replicate it in the methods section. With your recommendation now the protocol is easier to follow.

**• Line 179: Where is the annual incidence of Chagas cardiomyopathy sourced from? There is a wide range in the literature, so please include more references.**

We have added two new references, including a systematic review and meta-analysis. This ensures a more comprehensive and evidence-based estimate of the disease burden.

**• Line 181: Diagnosis and categorization of CCC also vary. Many newer articles include NYHA severity classes in the definitions, so please adjust to clarify this – you already touch on this in line 195.**

We acknowledge the importance of incorporating NYHA classification into our study. As a result, we have ensured that NYHA classification is clearly specified in Table 2, both for baseline assessment and follow-up evaluations, to facilitate a more standardized and clinically relevant classification of disease severity.

**• Line 253: Digestive tract involvement is less than 15%. Please correct. As you mentioned, some places in the Southern Cone have a higher proportion of digestive tract involvement, but this is not a generalization for Chagas. The Brazilian article identifies female sex as a predictor; however, this line could mislead readers. It is well known that only some genetic variants of T. cruzi are associated with digestive forms—this is the biggest association, not female sex.**

We have clarified that the figures of prevalence of digestive involvement refers to our setting, while also noting that rates are higher in certain regions of the Southern Cone. Additionally, we have streamlined the discussion on risk factors for digestive forms to maintain focus on the study’s objectives.

**• The methods section reads more like a review of the literature than a description of what the ChaNoE cohort will entail. It is important to be very detailed about your specific cohort, rather than existing literature. I see a review of the current recommendations for Chagas, but it’s not clear how the timeline works, who the team will consist of, how patients will be followed up, and how referrals will be made within the specific hospitals. Are these patients insured? Will they be using free national insurance? How will the team ensure that all follow-up care is completed? Will different sites use either benznidazole or nifurtimox for treatment, and how will that be decided? Based on the broad description, it sounds like these participants will be treated for all possible complications of Chagas disease.**

As mentioned above, we have modified the structure of the paper following your instructions. To clarify the commented issues (teams, hospitals, health insurance...) we have added some sentences to the section “The ChaNoE cohort”.

Patients with Chagas disease typically receive life-long follow-up care. Our objective is to maintain the cohort active for as long as possible; therefore, no predetermined conclusion date has been established. To minimize loss to follow-up, we have implemented proactive measures such as regular phone calls and email reminders to participants. These details have been incorporated into the text under the section “The ChaNoE Cohort.”

Regarding treatment, we have now explicitly stated that benznidazole is our first-line option. However, due to occasional supply constraints and specific patient-related considerations, we have allowed for the use of nifurtimox as an alternative.

Additionally, we have provided further clarification on the procedures for biobank creation, as well as the collection and storage of biological samples.

**• We don’t know the specific inclusion or exclusion criteria for participants, such as age ranges and eligibility. This is only described briefly in the abstract and not in detail.**

Inclusion and exclusion criteria are defined in “The ChaNoE cohort section. We aim to include any patient with chronic CD, with no age limits. We will only exclude acute cases and congenital cases if promptly treated. We have written it more clearly to facilitate its reading.

**• Line 331 says that patients will have easy access to their healthcare provider; however, it is unclear if this provider is part of the study or if it refers to a regular visit, with medical charts being reviewed.**

As part of the standard medical care provided to patients undergoing treatment, all participating centers have established specific pathways to ensure prompt medical attention in case of side effects. In some cases, these pathways include access to the emergency department, unplanned visits, or an emergency telephone service. Given the diverse operational structures of each center, we have included a general statement in this section to address these variations.

**Discussion:
• The discussion reads somewhat broadly about Chagas disease, rather than specifically discussing the cohort study in the context of previous work.**

We have revised certain sections of the discussion to emphasize the need for larger cohort studies, underscoring the importance of standardizing criteria and aligning them with the broader CD community to ensure comparability of results. We hope that these recent revisions address your concerns.

**Reviewer #3:**

**Dear Editor and Authors,**

**Thank you for the opportunity to review the manuscript titled "The Chagas Non-Endemic Cohort (“ChaNoE”): Aims and Study Protocol." This is an intriguing and valuable project. The manuscript effectively describes the establishment of a Spanish cohort designed to identify and monitor chronic cases of Chagas disease. The study employs a modern clinical-laboratory approach and clearly articulates its objectives and rationale in a well-justified manner. The manuscript addresses an important public health challenge: Chagas disease in non-endemic regions, a topic that remains underrepresented in current research. The focus on Spain—a country with a significant population of individuals living with Chagas disease due to migration—highlights the study’s relevance and potential impact. However, before the manuscript is accepted, several questions and concerns need to be addressed, as outlined below:**

**Major comments**

**1. The authors mention multiple times that the data obtained from the “ChaNoE” cohort will be compared with other cohorts worldwide. However, the manuscript does not specify these cohorts. Including examples of similar studies, such as Oxente Chagas Bahia, Samitrop, TESEU, and others, would enhance the manuscript by informing readers of relevant ongoing research efforts globally and providing context for the comparative analyses planned in this study.**

Thank you for your valuable comment. Upon reviewing the text, we realized that, as you pointed out, we had not referenced the main existing cohorts. Both the SamiTrop and the Oxente Chagas Bahia cohorts serve as exemplary models for Chagas disease research, and we have aimed to follow their approach. We have now included references to both cohorts in the Discussion section.

**2. While the protocol is thorough, some methodological aspects, such as the specific strategies for data standardization across centers, are underexplained. A clearer description of quality control for data and biobank management is needed.**

We have added a whole paragraph in the “Data management and statistical analysis” section explaining how data will be managed.

**3. The study’s reliance on multiple centers for participant recruitment could face logistical hurdles, especially in ensuring consistent diagnostic criteria and follow-up. Comment!**

This is an important limitation. The current protocol represents the first attempt to standardize the diagnosis, classification, and follow-up of patients living with CD in Spain. We acknowledge that there are certain gaps in our protocol, such as the methods used for serology or PCR, variability in the interpretation of cardiac imaging and electrocardiograms, and differences in the indication of other complementary exams across centers. However, we believe that these variations will not hinder the collection of meaningful and comparable data.

In response to your recommendation, we have added a paragraph in the Discussion section addressing these limitations.

**4. Although PCR is mentioned, its limited sensitivity in chronic cases raises concerns. The manuscript could better address how it will account for false negatives or inconsistent results. Explain how the study will mitigate the limitations of current diagnostic tools, such as PCR, and whether alternative or complementary methods will be employed.**

As you mention, the utility of PCR in the chronic phase is still a matter of debate. In this sense, we plan to assess its role during both diagnosis and follow-up to better understand its implications and dynamics, while acknowledging the limitations inherent in performing it according to the routine protocols of each center. Following recent clinical trials, such as MULTIBEZ, CHAGASAZOL, and FEXINIDAZOL, in which PCR was used as a marker of treatment failure, we will offer participants alternative treatment options if a positive PCR reappears during follow-up. This approach has already been implemented by all participating centers in recent years.

Validating the performance of the PCR method is beyond the scope of the present cohort, as each center employs variations of the technique in their real-world clinical practice. Should we decide to pursue this validation, we will propose a nested study within the cohort, with its own defined protocol and characteristics.

We have addressed these limitations and discussed the utility of PCR in the Discussion section.

**5. While the protocol outlines periodic evaluations, the manuscript does not provide sufficient detail on how adherence to follow-up will be ensured, particularly for participants in non-urban areas. Discuss strategies to overcome potential challenges in participant recruitment and retention, including communication methods and incentives.**

Reviewer 2 also raised this concern. Life-long follow-up is typically offered to patients with CD. In our setting, the vast majority of individuals with CD reside in urban areas, as these offer more opportunities for the migrant population, which contrasts with the situation in endemic regions like Brazil. Most patients are diagnosed through routine screenings conducted by primary care physicians, blood banks, or community events, and are then referred to specialized centers like ours as part of standard practice. No specific incentives are provided for participation. To minimize losses during follow-up, we proactively contact participants by phone or email when they miss scheduled visits. These explanations have been included in the section titled 'The ChaNoE cohort”.

**6. Inclusion criteria requiring diagnosis by two serological tests might exclude some cases, potentially limiting generalizability.**

This is a potential limitation, but we decided to follow the Spanish national recommendations for diagnosis. It already includes a third test if there are discordant results (see reference 10).

**7. Add visual elements, such as flowcharts or timelines, to illustrate the study design, recruitment process, and follow-up schedule.**

We have added the figure 1 that shows the general flowchart of participants through the cohort.

**8. The potential financial burden of implementing such protocols in other settings, including low-resource regions, is not addressed. Include a discussion of how the findings and methodologies could be adapted for low-resource or other non-endemic settings. In addition, provide examples of how the study outcomes will directly influence public health policies or clinical practices in non-endemic areas.**

Thank you for raising the issue of potential financial burden. While our protocol includes more complex tests such as PCR, MRI, and endoscopy, the core examinations consist of affordable complementary assays that are accessible in low-resource settings. We have included a specific explanation regarding this in the Discussion section (paragraph 4).

Regarding public health policies, we have addressed this in the fifth paragraph of the Discussion. One of the key objectives of the present study is to generate data that will inform evidence-based policies and management guidelines, which are directly applicable to our setting and can be adapted to similar contexts.

**9. Include plans for biobank management and data sharing policies.**
We have added more detailed information for the biobank management and data sharing policies in both sections as also recommended by reviewer 2.

**Minor comments**

**1. Line 44: Italicize Trypanosoma cruzi.**

Corrected.

**2. Line 49: Clarify the term "heterogeneous." As it stands, the meaning is unclear and could benefit from further explanation or rephrasing?**

We have modified the text to “Chronic Chagas cardiomyopathy (CCC) and digestive complications are the primary manifestations, yet diagnostic criteria, especially regarding organic involvement, and treatment indications are still a matter of debate”.

**3. Line 81: Remove the word "also." The phrase, as written, might imply that vectorial transmission occurs in non-endemic areas, which could lead to misinterpretation.**

Corrected.

**4. Line 403: Specify the microbiological tests used for diagnosing Chagas disease. Wouldn't these be more accurately described as parasitological tests?**

We agree that the proper term is parasitological test. We have also added a reference that exemplifies the shift of parasitological diagnosis.

**5. Line 470: Capitalize "Chagas."**

Corrected.
